# Governance Capability of the Public Health System: A Comparative Analysis of the Control of COVID-19 in the Different Provinces of China

**DOI:** 10.3390/ijerph18084210

**Published:** 2021-04-15

**Authors:** Yingfeng Fang, Fen Zhang, Chenyu Zhou, Ming Chen

**Affiliations:** School of Economics and Management, Wuhan University, Wuhan 430072, China; zhangfen@whu.edu.cn (F.Z.); younginwhu@outlook.com (C.Z.); effienol@hotmail.com (M.C.)

**Keywords:** public health, epidemic control, SEIR model, governance capability

## Abstract

At the beginning of 2020, the global outbreak of the novel coronavirus COVID-19 posed a huge challenge to the governance capabilities of public health in various countries. In this paper, the SEIR model is used to fit the number of confirmed cases in each province in China, and the reduction rate of the basic reproduction number is used to measure the actual score of the control effect of COVID-19. The potential capacity of prevention and control of epidemics, in theory, is constructed, and we use the difference between theoretical ability and actual score to measure the ability of governance of public health. We found that there were significant differences between actual effect and theoretical ability in various regions, and governance capabilities were an important reason leading to this difference, which was not consistent with the level of economic development. The balance of multiple objectives, the guiding ideology of emphasizing medical treatment over prevention, the fragmentation of the public health system, and the insufficiency of prevention and control ability in primary public health systems seriously affected the government’s ability to respond to public health emergencies.

## 1. Introduction

According to China’s national plan, 2020 is the final year to completely defeat poverty and implement a well-rounded strategy to build China into a society of moderate prosperity. However, the global outbreak of the novel coronavirus COVID-19 has brought considerable challenges to China’s economic development. In order to control the spread of the epidemic, various strategies were applied by the Chinese government, including large-scale quarantine, restrictions on travel, and the isolation and monitoring of suspected cases. It was a major test of governance capabilities to fight COVID-19, where the government was in desperate need to improve both the system of prevention and control during the epidemic and the management system of national public health emergencies.

In contrast to the continuous spread of the global epidemic, the local epidemic was controlled as early as the beginning of the outbreak in China, which reflects the strong governance capabilities of the Chinese government. Local governments spared no effort to carry out multichannels of publicity on the prevention and control of COVID-19, implementing joint prevention and control of units at all levels and giving full play to the advantages of the information networks of primary communities and village organizations to accurately lock onto their targets. However, the situation of global epidemic prevention and control is still not very good. Do governments have full use of their governance capabilities of public health emergency management systems? Why did the local governments have different levels of performance in the prevention and control of epidemic?

Therefore, we use the SEIR model to fit the data of confirmed cases at the provincial level and the reduction ratio of the basic reproductive number as the actual score indicator for epidemic control. At the same time, we take into account the medical resources, health resources, and population inflows of each province and then calculate the theoretical ability of epidemic control. Through a comparative analysis of the theoretical and actual capabilities of prevention and control, our paper first quantitatively evaluates the differences in the governance capabilities of local governments in the field of public health and then further discusses the influencing factors of governance capabilities and the existing problems in the construction of the public health system. 

Public health is defined as the science and art of preventing disease, prolonging life, and promoting health through the organized efforts and informed choices of society, organizations, public and private sectors, communities, and individuals [1]. It can be seen that the construction of public health cannot be separated from the extensive participation and joint efforts of all sectors of society. Inadequate prevention and control of governments facing public health emergencies can cause huge losses to people’s health and economic development. Barro et al. showed that the economic loss brought by influenza during the 1918–2020 outbreak was equivalent to 6–8% of a typical country’s GDP [2]. Eichenbaum et al. pointed out that people tend to reduce the severity of infectious diseases by cutting consumption and work, thereby worsening the extent of economic recessions caused by infectious diseases [3]. Jorda et al. concluded that pandemics had large negative macroeconomic effects, such as a decline in the return on assets [4]. Additionally, people’s social patterns, work patterns, and consumption patterns would all be affected by the impact of epidemics [5,6].

The reform of public health systems has been part of the development of various countries. When studying the causes of infectious disease epidemics in South Africa, Coovadia et al. pointed out that weak management, failed leadership, poor implementation of policies, and lack of human resources in the health sector greatly weakened the role of the public health system [7]. Potrafke found that government ideology and electoral motives influenced the effectiveness of public health systems [8].

In recent decades, China’s public health reform has made great achievements. People’s nutrition, drinking water and sanitation facilities, disease incidence, and medical security systems have all been improved. However, China’s public health system is still facing some new challenges. Gong et al. pointed out that the huge and accelerating population movement brought about by China’s urbanization had brought significant challenges to the urban public health system, including the increasing burden of disease, insufficient medical resources, and environmental pollution [9]. Compared with urban areas, inadequate and unbalanced allocation of rural health institutions were also made more prominent. The teams of rural doctors were even more worrying for the following reasons: their professional capabilities were very low, and the proportion of full-time doctors was very small; low income levels made rural health institutions unattractive to medical university graduates, and the efficiency of use of rural public health funds needed to be improved [10,11].

In summary, a good public health system and strong government governance capabilities are important guarantees for national health and economic development, as well as an important support system for responding to public health emergencies. Among them, strong government governance is a necessary condition for the public health system to play an effective role. However, existing literature lacks normative research on governments’ governance capabilities of public health systems, and there are few quantitative analyses. This article is based on a comparative analysis of the theoretical and actual capabilities of various regions in China in the prevention and control of COVID-19; it provides some valuable experiences for epidemic control in other countries around the world.

The paper is organized as follows. Section 2 describes the data and model. Section 3 presents the results of governance capability. Section 4 documents further discussions on the governance capability of the public health system. Section 5 concludes.

## 2. Data, Models, and Analysis of COVID-19 Transmission

### 2.1. Data

The data of COVID-19 used in this paper was collected from the circulars of national and provincial health commissions, which included confirmed and suspected COVID-19 cases, and the daily circulars of official media of each province on the tracked activity of confirmed COVID-19 patients. As of 24:00 h on 5 March 2020, a total of 80,585 cases of COVID-19 had been diagnosed nationwide; 3016 cases had died, and a total of 52,416 cases had been cured; 25,153 cases were confirmed, and 522 cases were suspected at that time. The data on migration came from the migration trend function of the Baidu Epidemic Map, and we selected the top ten provinces, with the exception of Hubei Province, including Henan, Hunan, Anhui, Jiangxi, Guangdong, Chongqing, Jiangsu, Sichuan, Shandong, and Zhejiang. The specific numbers of the migrant population are shown in Table 1. 

### 2.2. Propagation Dynamics Model

There are lots of propagation dynamics models, including SI, SIR, SEIR, that are used to simulate epidemic situations of infectious diseases in the existing literature, where S, E, I, R mean susceptible, exposed, infected and removed, respectively. These models were used to predict the trend of the number of cases nationwide with national data or Wuhan data. However, the time and number of cases appearing in each province were quite different, which is problematic to a large extent. For example, the first patient in Wuhan appeared about a month and a half earlier than the first patient in other regions. In this context, predicting the epidemic data and turning points on a national basis is an unreasonable proposition. In addition, there are significant differences in the medical resources, population migration, and government governance capabilities of various provinces. In response to these problems, this paper chooses to use the SEIR model to predict the spread of the COVID-19 epidemic in various provinces and uses the comparison between the predicted results and the actual situation to evaluate the capabilities of local epidemic control. 

The SEIR model is based on the basic framework of the SI model and takes into account the dynamic changes of the patient population. The population is divided into S (Susceptible), I (Infected), R (Removed, the number of individuals cured or dead), and those in incubation period E (Exposed). In this way, the population size N satisfies the following equation:

(1) N(t)=S(t)+I(t)+R(t)+E(t)

Assume that susceptible individuals come into contact and become infected individuals, with probability *β*. *S/N* represents the proportion of susceptible individuals, and the susceptible population will decrease according to the following rate of change:

(2) $$\frac{dS}{dt}=-\frac{\beta SI}{N}$$

At the same time, assume that patients in the incubation period turn into an infected person with probability $\gamma_{1}$; the dynamic equation for patients in the incubation period is

(3) $$\frac{dE}{dt}=\frac{\beta SI}{N}-\gamma_{1}E$$

The increase in the number of infected people I comes from the diagnosis of the exposed population E, and, at the same time, I transforms to R with probability $\gamma_{2}$; then, the dynamic equation of the infected I and the removed E are as follows:

(4) $$\frac{dI}{dt}=\gamma_{1}E-\gamma_{2}I$$

(5) $$\frac{dR}{dt}=\gamma_{2}I$$

This paper uses Python to simulate the epidemic and fit transmission parameters, including transmission probability and basic reproduction number, and then substitutes them into the following epidemic prediction, where the basic reproduction number (R0=βγ2) means the number of secondary infections produced by a single infective person in a susceptible population. 

### 2.3. Prediction 

Considering the incubation period of COVID-19 ranges from 1 to 14 days, we chose an average incubation period of 7 days and set the probability of an individual from incubation state E to infection state I as 1/7. After that, we explore the emergency measures of the provincial governments and the role of the public medical system in epidemic control. Therefore, we will make predictions for each province. However, it will bring about two problems: first, before the emergence of second-generation infectious cases, the growth of confirmed cases does not truly reflect the parameters of the spread of novel coronavirus. Mistaking imported cases as transmissible cases will lead to overestimation of the probability of infection. Second, the number of cases in most provinces was small, and reasonable predictions cannot be made when the sample size is insufficient.

Considering these two problems, our paper used the data from Wuhan to fit the parameters of the SEIR model. As Wuhan Municipal Health Commission did not promptly notify the changes in confirmed cases from 1 January 2020 to 18 January 2020, we could not obtain the specific transmission trend of COVID-19 in the first half of January 2020; only case data after 19 January could be obtained. Based on the existing public information, we could roughly conclude that the citizens would not have known the seriousness of the situation of the novel coronavirus before 20 January 2020. This also means that the spread of the virus before 18 January 2020 reflected the truest transmission capacity in a natural state. We regarded 8 December 2019, which is considered to be the time of discovery of the first patient with novel coronavirus pneumonia in China, as the starting point for model analysis. Regarding 19 January 2020 as the 42nd day of when the epidemic began to spread and 23 January 2020 (the shutdown of Wuhan) as the 46th day, we then fitted the parameters of the SEIR model. By calculation, the propagation probability was 0.04, and the basic reproduction number $R_{0}$ was 3.7.

**Figure 1:**
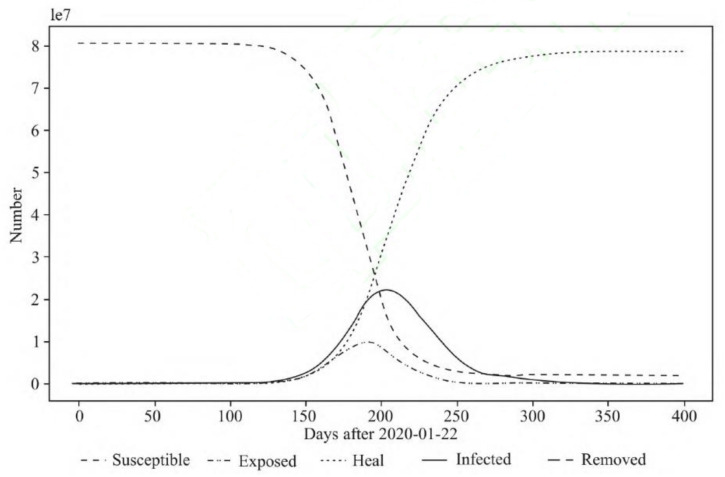
Transmission trend of COVID-19 in Jiangsu Province.

**Figure 2:**
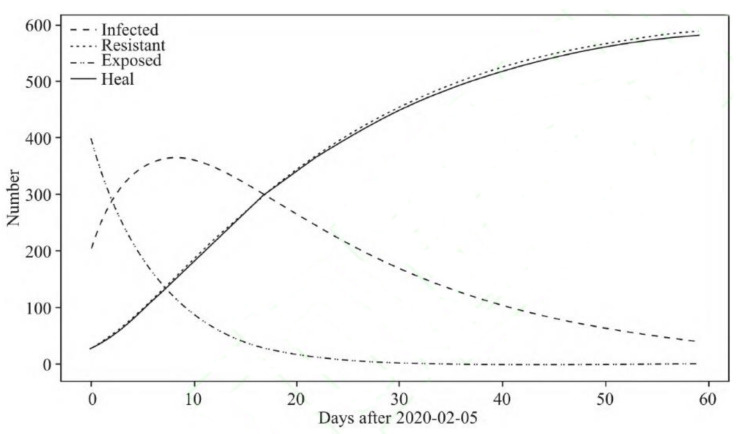
Transmission trend of COVID-19 in Jiangsu under ideal conditions.

The spread parameters obtained by fitting Wuhan data were used to simulate the development of the epidemic situation in various provinces and cities, as shown in Table 1. Taking Jiangsu Province as an example, the first case of second-generation transmission in Jiangsu Province appeared on 30 January 2020. Therefore, the 168 cumulative cases before 30 January were regarded as imported cases and 22 January 2020 was set. The parameters were 1 infected person and 167 latent persons. The simulation results are shown in Figure 1 and Figure 2. According to Figure 1, the turning point will come about 180 days after 22 January 2020, but the actual situation was that the growth of cases in Jiangsu Province was under control by 6 March 2020, and there are no new confirmed cases for 16 consecutive days. Obviously, the spread of the novel coronavirus pneumonia had been effectively controlled.

The specific results of other provinces and cities are shown in Table 2. It can be seen that without any intervention, the turning point of the epidemic in all provinces will appear in 170–200 days; the cumulative infection rate of residents in each province will exceed 80%, and the maximum simultaneous infection rate will be 20–25%. In fact, as of 6 March 2020, the epidemic in all provinces and cities in China had been effectively controlled, and most new cases reached zero or single digits. This reflects the effective measures of China’s response to the epidemic (due to the complex situation abroad, imported cases from abroad are not considered).

As Table 2 shows, there is a big gap between the ideal control result and the actual situation. In practice, a few days are taken to reach the turning point in various provinces—the more days needed to reach the turning point under ideal control conditions, the worse the actual effect of prevention and control in the epidemic. The same applies to the analysis of the maximum number of infections. Taking Jiangsu Province and Zhejiang Province as examples, without policy intervention, the turning point of Jiangsu requires 184 days, and the number of infected people would reach 21.46 million, while the turning point under ideal control is 22 days, and the maximum number of infected people is 334. The actual turning point was reached in 24 days, and the actual maximum number of infections was 456. Compared with Jiangsu Province, epidemic control in Zhejiang was a little worse. Without policy intervention, the turning point of Zhejiang would take 175 days, and the number of infections would reach 22.61 million, while the turning point under ideal control is 19 days, and the maximum number of infections is 688. In reality, it did not reach the turning point until the 23rd day, and the actual maximum number of infected people reached 921. The gaps in various provinces may be caused by differences in the efficiency of medical resource utilization and government governance capabilities. This will be measured and evaluated in the following sections.

## 3. Results: Government Governance Capacity and Epidemic Control

**Figure 3:**
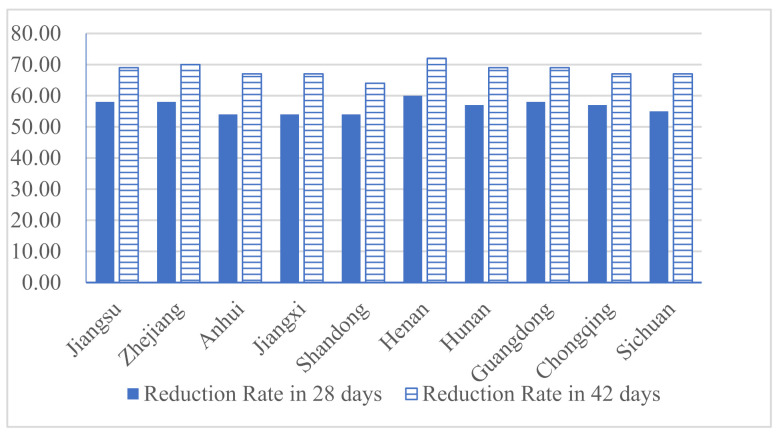
The reduction rate of the basic reproduction number R0.

The paper uses the reduction rate of the basic reproduction number $R_{0}$ as a measure of the degree of epidemic control. The main problem we faced with this operation is that most of the cases in the early stage (before 31 January 2020) were imported into each province or city rather than being infected. Considered this problem, we chose to set the confirmed cases before 31 January as exposed on 21 January, which can greatly reduce the error caused by treating imported cases as second-generation transmission cases. Considering that most of the incubation periods of the disease do not exceed 14, 28, and 42 days (2 and 3 incubation periods), these periods are selected as cycles to observe the reduction in $R_{0}$. The results are shown in Figure 3. On the whole, the provinces with a large imported population from Wuhan have done a better job in the prevention and control of COVID-19. In 42 days, the reduction ratio of the basic reproduction number reached almost 70%.

Additionally, Figure 3 also shows that regardless of whether it is in two incubation periods or three incubation periods, there are obvious differences in the results of epidemic control in the various provinces and cities. Combining existing relevant literature [2], we believe that factors such as economic development, medical and health resources, government governance capabilities, and public health behaviors are likely to be the causes of the differences. Therefore, our paper creates an indicator to reflect the differences in local governance capabilities. Specifically, first, the model uses the reduction rate in the basic reproduction number $R_{0}$ to reflect the actual response capacity of the local government to the epidemic $R^{R}$, $R^{R}=\Delta R_{0}/R_{0}$. Second, we use the medical and health resources of various provinces and cities, migration population, and other factors to construct the theoretical response capacity $R^{T}$. Finally, we use the standardized ratio of $R^{R}/R^{T}$ to reflect the government’s governance capacity in the field of public health. The larger the ratio, the more efficient the local government’s allocation of medical and health resources and the stronger the ability to prevent and control emergencies.

This paper considers the comprehensive measurement of the amount of medical and health resources in each province to express the theoretical endurance of the province’s public health system *M*, taking the imported population from Wuhan as pressure *P*. Therefore, in the face of public health emergencies, the theoretical response capacity of each province can be calculated by the following formula:

(6) $$R^{T}=M/P$$

Among them, theoretical endurance is an indicator constructed using the current public health resources of each province:

(7) $$M=\sum_{i}W_{i}X_{i}$$

The per capita values of the relevant indicators shown in Table 3 are linearly standardized and summed up, namely

(8) $$X_{i}=\sum_{i}w_{ij}X_{ij}$$

Among them, $W_{i}$ and $w_{ij}$ are the weights. For the sake of simplicity, we set equal weights for $W_{i}$. According to the existing literature, corresponding weights are assigned according to the relative importance of various indicators for $w_{ij}$, and each indicator is standardized to the interval [0, 100]. The data used to measure the theoretical prevention and control capabilities of each province in Table 3 are from the China Health Statistics Yearbook 2019. The indicators mainly include the degree of regional economic development, public health expenditure, the public health system, and medical resources (including the number of hospitals and doctors). The number of hospitals and doctors reflects the ability to treat patients, the number of beds reflects the ability to treat and isolate patients, and the number of professional public health institutions (including CDC) reflects part of the ability to track patient trajectories.

**Figure 4:**
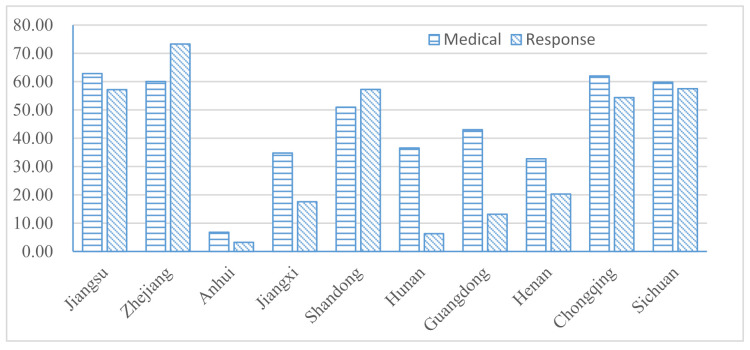
Scores of theoretical medical endurance and response capacity in each province.

**Figure 5:**
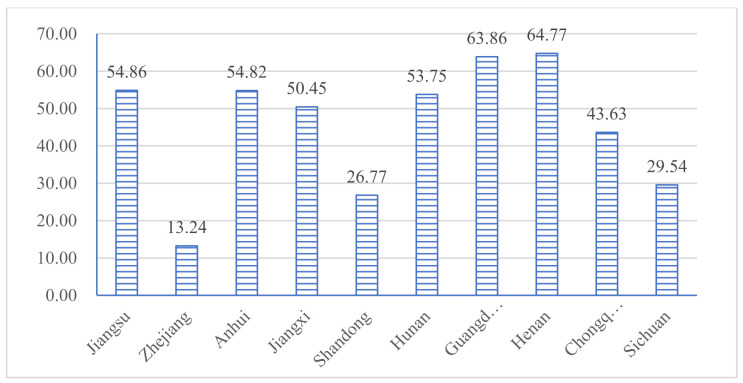
Scores of governance capacity for each province.

The results of theoretical endurance *M* and theoretical response capacity $R^{T}$ are shown in Figure 4. It can be seen, intuitively, that Zhejiang Province has the strongest theoretical response capacity and Anhui Province has the weakest. The result of the ratio, which is used to represent the government’s governance capacity, is shown in Figure 5. The order of governance capacity of each province is as follows: Henan, Guangdong, Jiangsu, Anhui, Hunan, Jiangxi, Chongqing, Sichuan, Shandong, and Zhejiang. We find that the size of governance capacity is not necessarily positively correlated with the degree of economic development. Henan Province, in central China, ranks first, followed by Guangdong and Jiangsu; these provinces have higher capabilities in prevention and control. However, the scores of Shandong and Zhejiang are relatively low in governance capabilities among these 10 provinces. 

Why are the scores so different from our intuited scores? We attempt to answer this question from two aspects of government governance capabilities—cognitive ability and executive ability.

The primary reason is a lack of cognitive ability. The local government had a lack of willingness to seek help from intellectuals; the semiclosed management pattern hindered the rational deployment of knowledge and talents and the effective integration of resources, keeping various resources in a highly separated state. When this kind of governance structure encounters public health emergencies, the lack of cognitive ability will limit the government’s ability to execute any plans. Specifically, the government puts too much emphasis on its own strong execution ability and neglects the improvement of cognitive ability. Wuhan is one of the big cities in China with numerous higher education institutions. It has the only national level-4 biosafety laboratory (P4 laboratory) in the country, and the public health program of Huazhong University of Science and Technology ranks A+. However, due to weaker government cognitive capabilities, the high-quality resources could not play an important role. Moreover, on the eve of the World Military Games, Wuhan Customs also simulated the entire process of handling a case of novel coronavirus infection found in an airport channel and performed the exercise with an epidemiological investigation, medical investigation, temporary quarantine area setting, and isolation, case transfer, and sanitation treatment. This also reflects that if the Wuhan government had recognized the seriousness of the epidemic in the early stage of the epidemic, Wuhan had sufficient capacity and resources to stunt COVID-19 in its infancy.

As evidence of the importance of cognitive ability, let us look at the other two provinces that performed extremely well in the epidemic, Guangdong and Henan. 

First of all, the lessons from SARS made Guangdong one of the first provinces in the country to respond; the official government could give clear professional epidemic prevention guidance while focusing on calming people’s emotions. The more balanced information content, coupled with the release time of official news (which was significantly ahead of the country), placed the control of COVID-19 in Guangdong on a different path from SARS 17 years ago. Thus, the quality of information obtained by the citizens also varied greatly. The public effectively cooperated with the emergency control measures issued by the government. Obviously, the experience of SARS in Guangdong had greatly improved the cognitive capabilities of the Guangdong government, allowing the government to communicate with disease control agencies and medical institutions smoothly, cooperate with them closely, and respond extremely quickly to the epidemic.

Secondly, the performance of Henan province in the prevention and control of the epidemic exceeded the public’s expectations. Both the results from this article and the “hard-core” epidemic prevention measures indicate that the Henan government had strong governance capabilities that did not match its weaker economic development and larger population inflows. According to the official media outlet Henan Daily, on 1 February 2020, Henan stopped the shuttle buses from Zhengzhou to Wuhan as early as the end of December 2020. On 21 January, the sale of live poultry was banned. On 22 January, 130 designated hospitals for the medical treatment of pneumonia infection by the novel coronavirus were announced. On 23 January, people returning from Wuhan were urged to report to their village and neighborhood committees in a timely manner and stay at home for 14 days. On 24 January, a conference on prevention and control at work was held. Obviously, Henan Province was aware of the huge risks hidden behind the epidemic as early as the end of December. Such cognitive ability can be said to be very good.

The second aspect of government governance capacity is execution capacity. As a matter of fact, the execution capabilities of all provinces in this epidemic were excellent; the deployment of resources and epidemic prevention measures were timely and effective after recognizing the severity of the epidemic. From this, we can simply infer that the government’s cognitive ability limited its execution ability, thereby reducing the government’s comprehensive governance ability. It is noted that the lack of cognitive ability has become a restriction to modernizing Chinese governance. To improve the governance capabilities of governments at all levels, it is necessary to restrain the arrogance of power, give up the belief in arbitrary execution capabilities, take practical measures to strengthen the circulation of knowledge with professional institutions, and promote the transformation of governance methods.

## 4. Governance Capacity and Public Health: Further Discussion

COVID-19 is undoubtedly a considerable challenge to the governance capabilities of the public health systems in China and all over the world. From the results, with the strong control of governments at all levels, China’s public health system withstood the test and controlled the epidemic. However, we have not done well enough, especially in the early stage of the outbreak. What prevented the local governments from fighting the epidemic with a more complete and effective public health system? 

First, the tradeoff of local government’s targets. Governments at all levels are faced with a tradeoff between multiple goals, such as economic development and public health improvement. In the long run, as an important part of human capital, health is an important engine for economic development. The improvement of health has a positive impact on both labor productivity and economic growth [12]. However, in the short term, an increase in public health spending will have a crowding-out effect. On the one hand, private health investment will be crowded out, and the government’s financial pressure will be increased. On the other hand, it will crowd out other public infrastructure investments. The choice of government officials to develop the economy or improve public health depends on the assessment and promotion system. Under the political competition system, with GDP as the assessment target, GDP improvement has become the core goal of government officials, and public health may become a victim. In recent years, the central government has downplayed the assessment of GDP; hence, the political championship centered on economic growth has undergone a series of adjustments [13]. However, in 2017, the proportion of public health expenditure to GDP was still at a low level—the proportion basically lies within 8%, which was much lower than the level of the major developed countries and even some developing countries during the same period (shown in Table 4). Although public health expenditure accounted for a relatively higher proportion in the United States and some European countries, they did not perform well in the control of COVID-19. The reason for that was their leaders’ strategies of prevention and control were inappropriate, and they missed the best time for the prevention and control of the epidemic. Additionally, there are also some deeper reasons, such as institutions and culture. Public health services have significant positive externalities. Therefore, local governments cannot underestimate the role of investment in public health, no matter whether they are based on the “quasi-public goods” attribute of public health resources or the development strategy of “Healthy China”.

Second, the guiding ideology of emphasizing medicine over prevention. China’s medical and health system has prioritized medical care over health for a long time, with hospitals as the focus, and service provision is fragmented. Compared to the average of 38% in OECD countries, expenditures in hospitals account for 54% of China’s total health expenditures (World Bank Report, 2016). Yip et al. also pointed out that China’s ten-year medical reform, from 2009 to the present, basically focused on medical provisions but ignored public health. Public health is an important factor in health [14]. It not only affects the health of individuals but also the health of the entire society. China has invested a lot of manpower and material resources in the ten-year medical reform and established an online direct reporting system for statutory infectious diseases. However, it was almost useless at the beginning of the COVID-19 outbreak. The proportion of health personnel in disease control agencies has also dropped from 2.53% in 2009 to 1.53% at present. The development strategy of “Healthy China” clearly focuses the guiding ideology of public health on prevention. However, in actual situations, due to the large gap in salaries and investments between hospitals and health institutions, it is difficult to implement the corresponding mechanisms. Public health has a public welfare attribute, which is different from the paid medical services of hospitals. 

Third, the fragmentation of public health governance. The mismatch of functions, capabilities, and powers of the public health system at all administration levels has led to the fragmentation of government governance in public health. China’s CDCs, at all levels, are under vertical management in terms of professional guidance, while executive leadership management is horizontal. The higher level CDC has no personnel and financial rights over the lower level CDC and can only arrange work without funding and wages, resulting in lower management efficiency and being unable to quickly respond to public emergencies [15]. The medical and health industry involves more than a dozen government departments, and they are all committed to achieving their respective goals. Therefore, there are coordination problems between health institutions at all levels and local governments; they lack effective communication mechanisms and even have mutual competition and exclusion, which increases the overall cost of prevention and control of disease and hinders the process of medical and health reform.

In addition, the emergency response mechanism requires governors to have a biomedical background for major incidents of public health. Under the current public health system, with inconsistent powers and responsibilities, the management personnel of local departments in disease control are mainly arranged by local governments—most of them are nonprofessionals. Therefore, as a public health governor, there is neither sufficient relevant knowledge nor sufficient cognition to be in the role of professionals. After the SARS epidemic ended in 2003, the World Health Organization organized a team of experts from China and other countries to trace the origin and early transmission of the SARS virus. At that time, 7 of the 8 experts in the international experts’ group had a background in veterinary medicine or animal health, while only 1 of the 6 experts in China had this background [16].

**Figure 6:**
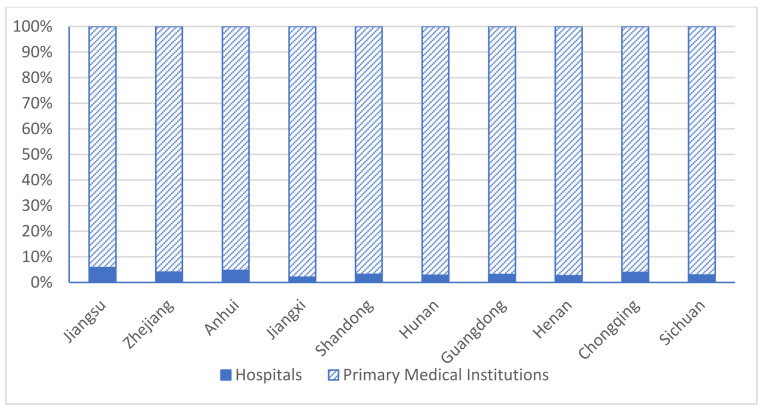
Total number of hospitals and primary medical institutions. Data source: China Health Statistics Yearbook 2018.

Finally, the capabilities of the public health system in prevention and control at the primary level are insufficient. As the primary public health system is in a special position at the forefront of prevention and control, it is the key to handling public health emergencies. Communities and village committees have obvious advantages as information networks and can accurately and quickly discover various neighborhood problems. Although the total number of medical and health personnel in China has increased in the past decade, it is still difficult for primary health institutions and poor rural areas to attract and retain qualified medical personnel. The proportion of primary health personnel in healthcare teams has dropped from 40% in 2009 to 36% in 2013 (World Bank Report, 2016). Moreover, the huge difference in the service quality of China’s primary health institutions also prevents patients from seeking treatment in primary institutions [17]. Most primary health technicians have low education, lack diagnostic capabilities, and have very limited knowledge of how to deal with infectious diseases, which make the public distrust the capabilities, knowledge, and information of these primary health personnel [18]. However, in contrast to the weakness in capability of primary prevention and control of public health, primary medical and health institutions are a major part of the entire medical and health system. As shown in Figure 6, of the total number of hospitals and medical institutions in each province, primary medical institutions account for more than 95%, far exceeding the total number of hospitals at all levels. Therefore, it is necessary to strengthen primary capabilities in prevention and control in rural areas and communities.

## 5. Conclusions

Through comparative analysis of theoretical control capabilities and actual potential prevention of various regions, we found that, first, inadequate governance capabilities are an important factor that hinders the public health system from exerting its maximum capabilities in prevention and control. Governance capability is mainly restricted by cognitive ability. The government’s powerful execution ability is difficult to fully exert under the influence of insufficient cognitive ability. Therefore, in order to improve the governance capabilities of the public health system, the most urgent task is to improve cognitive ability, which needs to adjust the evaluation system of local officials, balance the goals between economic growth and public health, clarify the rights and responsibilities of public health departments, and improve the fragmentation of management of the public health system. At the same time, the government should strengthen knowledge communication with universities and professional institutions, promote the transformation of governance methods, and realize the modernization of governance in the field of public health.

Second, transparent information communication is also a necessary means of governance. A modern public health system requires governments at all levels to make full use of modernized media, strengthen the dissemination and communication of health information, and increase public awareness in health and prevention. At the same time, it must strengthen the legal construction of information dissemination, increase the transparency of information dissemination, and give full play to the positive propaganda role of new media. With regard to the development of science and technology, government departments should also upgrade their information dissemination channels to achieve better results. 

Finally, because China’s public health system has placed too much emphasis on medical care and neglects the construction of the primary public health system, the primary healthcare system has insufficient medical capacity and lacks credibility. Therefore, in future medical reform, it is necessary to pay more attention to the primary healthcare system, strengthen the training of primary health technicians, and improve the coordination among various departments in the public health system. In terms of the allocation of health institutions, the structure of health personnel, and the performance and personnel arrangements of the healthcare system, China should build a viable incentive mechanism for the primary public health system to attract outstanding talents to the primary health system and construct a three-dimensional primary prevention and control system, with multiple collaborations between primary community leaders, health personnel, and residents.

## Figures and Tables

**Table 1:** Population migration from Wuhan before the lockdown (unit: %).

		20 January	21 January	22 January	23 January
To					
Henan Province		6.22	6.18	5.67	5.32
Hunan Province		3.36	3.40	3.24	3.07
Anhui Province		2.27	2.27	2.10	1.92
Jiangxi Province		2.09	2.04	1.95	1.84
Guangdong Province		1.66	1.69	1.56	1.55
Chongqing Province		1.27	1.25	1.04	1.00
Jiangsu Province		1.26	1.16	1.03	0.95
Sichuan Province		1.21	1.13	0.97	0.83
Shandong Province		1.03	1.00	0.85	0.69
Zhejiang Province		0.99	0.89	0.74	0.66

**Table 2:** The prediction of the SEIR model in various provinces of China.

Provinces	Days Required for Turning Point without Any Policy Intervention	Days Required for Turning Point under Ideal Control	Actual Days Required for Turning Point	Maximum Number of Infections without Policy Intervention (Million)	Maximum Number of Infections under Ideal Control	Actual Number of Infections
Jiangsu	184	22	24	21.46	334	456
Zhejiang	175	19	23	22.61	688	921
Anhui	180	21	22	18.12	527	777
Jiangxi	191	20	23	9.09	518	712
Shandong	198	22	30	26.84	284	474
Hunan	188	19	20	18.84	656	698
Guangdong	192	18	19	28.93	917	1007
Henan	193	18	19	31.25	775	901
Chongqing	171	20	22	8.00	334	423
Sichuan	202	18	22	21.45	314	356

Note: ① The maximum number of infections refers to the highest number of existing confirmed cases. ② No policy intervention refers to the extreme situation where the government does nothing and allows the epidemic to develop; ideal control refers to the result of the government giving full play to the existing medical and health system to its fullest effect. In the model, it is assumed that every patient can be treated in time. With isolation, the number of daily close contacts of infected person I is set to 0–0.5.

**Table 3:** Economic development and medical resources.

Indicator Variables	Name
Economic development	GDP per capita (10,000 yuan)
Public expenditure	General budget public expenditure per capita
	Public health expenditure per capita
Medical institutions	Number of general hospitals per capita
	Number of other hospitals per capita
	Number of primary healthcare institutions per capita
	Number of professional public health institutions per capita
Health staff	Number of health workers per capita
	Number of health technicians per capita
	Number of licensed physicians per capita
	Number of health workers in primary healthcare institutions per capita
Hospital beds	Number of hospital beds per capita
	Number of beds in primary healthcare institutions per capita

**Table 4:** Comparison of health expenditures in major countries in the world in 2015.

Country	Out-of-Pocket Medical Expenditure (% of Total Medical Expenditure)	Insured Medical Expenditure (% of Total Medical Expenditure)	Total Medical Expenditure (% of GDP)
China	40.22	59.78	5.32
German	15.53	84.47	11.15
France	21.08	78.92	11.07
England	19.64	80.35	9.88
India	73.52	25.59	3.89
Japan	15.88	84.12	10.84
Korea	43.60	56.40	7.39
Russia	38.92	61.08	5.56
Singapore	48.12	51.88	4.25
America	49.64	50.36	16.84

Note: The data in the table come from the World Bank database and was collected by the author.

## Data Availability

The data that support the findings of this study are openly available in National Health Commission of the People’s Republic of China and National Bureau of Statistics of China.
